# 

**Published:** 1911-07

**Authors:** 


### CONTENTS.

ORIGINAL COMMUNICATONS—
Anatomy of the Ileum, Intestinal Glands and Spleen. By Dr. J. F. Grant, Ph.B., M. D., Atlanta ........ 171
Clinical and Differential Diagnosis in Typhoid Fever. By Cyrus W. Strickler, M. D., Atlanta, Ga. ........ 177
Laboratory Findings in Typhoid. By J. Edgar Paullin, B. A., M. D. ........ 180
The Pathology of Typhoid Fever.. By E. Bates Block, M. D., Atlanta, Ga. ........ 185
The Valuation of Some Enteric Fever Symptoms. By Rufus T. Dorsey, B. S., M. D. ........ 191
The Diagnosis and Treatment of Chronic Bright's Disease. By Lewis M. Gaines, B. S., M. D., Atlanta, Ga. ........ 198
A Further Report on the Treatment of Pellagra. By Geo. M. Niles, M. D., Atlanta, Ga. ........ 214
EDITORIALS ........ 218
NEWS AND NOTES ........ 219
BOOK REVIEWS ........ 223
MEDICAL ITEMS ........ 225

